# Impact of antibiotics on gut microbiome composition and resistome in the first years of life in low- to middle-income countries: A systematic review

**DOI:** 10.1371/journal.pmed.1004235

**Published:** 2023-06-27

**Authors:** Charlie C. Luchen, Mwelwa Chibuye, Rene Spijker, Michelo Simuyandi, Caroline Chisenga, Samuel Bosomprah, Roma Chilengi, Constance Schultsz, Daniel R. Mende, Vanessa C. Harris

**Affiliations:** 1 Amsterdam UMC, location University of Amsterdam, Department of Global Health, Amsterdam Institute for Global Health and Development, Amsterdam, the Netherlands; 2 Research Division, Centre for Infectious Disease Research in Zambia, Lusaka, Zambia; 3 Amsterdam Institute of Infection and Immunity, Infectious Diseases, Amsterdam University Medical Center, Amsterdam, the Netherlands; 4 Department of Biostatistics, School of Public Health, University of Ghana, Accra, Ghana; 5 Zambia National Public Health Institute, Ministry of Health, Lusaka, Zambia; 6 Republic of Zambia State House, Lusaka, Zambia; 7 Amsterdam UMC, location University of Amsterdam, Department of Medical Microbiology, Amsterdam, the Netherlands; 8 Amsterdam UMC, location University of Amsterdam, Department of Internal Medicine, Division of Infectious Diseases, Amsterdam, the Netherlands

## Abstract

**Background:**

Inappropriate antimicrobial usage is a key driver of antimicrobial resistance (AMR). Low- and middle-income countries (LMICs) are disproportionately burdened by AMR and young children are especially vulnerable to infections with AMR-bearing pathogens. The impact of antibiotics on the microbiome, selection, persistence, and horizontal spread of AMR genes is insufficiently characterized and understood in children in LMICs. This systematic review aims to collate and evaluate the available literature describing the impact of antibiotics on the infant gut microbiome and resistome in LMICs.

**Methods and findings:**

In this systematic review, we searched the online databases MEDLINE (1946 to 28 January 2023), EMBASE (1947 to 28 January 2023), SCOPUS (1945 to 29 January 2023), WHO Global Index Medicus (searched up to 29 January 2023), and SciELO (searched up to 29 January 2023). A total of 4,369 articles were retrieved across the databases. Duplicates were removed resulting in 2,748 unique articles. Screening by title and abstract excluded 2,666 articles, 92 articles were assessed based on the full text, and 10 studies met the eligibility criteria that included human studies conducted in LMICs among children below the age of 2 that reported gut microbiome composition and/or resistome composition (AMR genes) following antibiotic usage. The included studies were all randomized control trials (RCTs) and were assessed for risk of bias using the Cochrane risk-of-bias for randomized studies tool. Overall, antibiotics reduced gut microbiome diversity and increased antibiotic-specific resistance gene abundance in antibiotic treatment groups as compared to the placebo. The most widely tested antibiotic was azithromycin that decreased the diversity of the gut microbiome and significantly increased macrolide resistance as early as 5 days posttreatment. A major limitation of this study was paucity of available studies that cover this subject area. Specifically, the range of antibiotics assessed did not include the most commonly used antibiotics in LMIC populations.

**Conclusion:**

In this study, we observed that antibiotics significantly reduce the diversity and alter the composition of the infant gut microbiome in LMICs, while concomitantly selecting for resistance genes whose persistence can last for months following treatment. Considerable heterogeneity in study methodology, timing and duration of sampling, and sequencing methodology in currently available research limit insights into antibiotic impacts on the microbiome and resistome in children in LMICs. More research is urgently needed to fill this gap in order to better understand whether antibiotic-driven reductions in microbiome diversity and selection of AMR genes place LMIC children at risk for adverse health outcomes, including infections with AMR-bearing pathogens.

## Abstract 2

Vanessa C Harris and colleagues review the currently available literature to report how antibiotic use in children from low- and middle-income countries impacts the gut microbiome and, the implications for antimicrobial resistant infections.

## Author summary

**Why was this study done?:**

- Antibiotics can disturb the bacteria in the gut and drive the development of antimicrobial resistance (AMR). Children in low- and middle-income (LMIC) settings often receive (inappropriate) antibiotics and are at risk of antimicrobial-resistant infections that are harder and costlier to treat.
- Currently, little knowledge exists regarding the risks of antibiotic use in children in LMIC and the impact of antibiotics on the bacteria in their gut.
- In this review, we aimed to identify studies evaluating the impact of antibiotics in children in LMIC to better describe these unintended antibiotic side effects.

**What did the researchers do and find?:**

- We systematically collected studies performed in LMIC in children that reported the effects of antibiotics on gut bacteria and profiled their antibiotic resistance genes.
- Our search revealed a paucity of published studies focusing on this topic and we identified only a handful of eligible studies to include in the review. We compared these studies based on the antibiotic used and differential impacts on gut bacteria, i.e., gut microbiome profiles and/or resistance genes.
- We observed that among children in LMIC, antibiotics generally reduced the numbers of bacterial taxa in the gut and increased the number of bacterial taxa with AMR.

**What do these findings mean?:**

- Our study shows that antibiotic use alters the microbiome composition in the gut and may adversely impact the health of children in LMIC, e.g., by placing them at risk of antimicrobial-resistant infections.
- More research is needed to understand how different antibiotics alter the gut microbiome and resistome of children in LMIC.
- Antibiotic usage in these populations should be better regulated, in order to prevent these indirect impacts of antibiotic use and reduce the risk of antimicrobial-resistant infections.

## Introduction

Bacterial antimicrobial resistance (AMR) is an already present and growing threat to global health and economies [1,2]. Low- to middle-income countries (LMICs) are disproportionately burdened by AMR [3], with some of the highest attributable mortality rates [4] and the least resources to control AMR, resulting from limited laboratory capacity, surveillance systems, and health care infrastructure. The World Health Organization (WHO) has identified AMR as a critical threat to already strained health systems, food security, and development in LMIC regions [5].

Human antibiotic consumption is a key driver of AMR, which arises when microbes become resistant to antimicrobials [6–8]. Antibiotic consumption is rising and distributed inequitably across geographic scales [9]. Children in LMICs have high frequencies of infectious disease episodes and a great need for antibiotic access, yet are also the most vulnerable to AMR and the risks of appropriate and inappropriate antibiotic consumption [10,11]. There is insufficient knowledge on how antibiotic consumption may place children in LMICs at risk for adverse health impacts and AMR.

Antibiotics, when used appropriately, reduce all-cause mortality and are lifesaving for key infectious diseases among children in LMICs [12,13]. Yet, they are frequently used inappropriately [14] and have multiple off-target effects in human hosts, with specific, under-characterized risks for children in LMIC settings. Antibiotic exposures significantly alter the short- and long-term maturation of the intestinal microbiome in children [15–17]. How these alterations differ by antibiotic class and translate into clinical benefits or risks of disease in different populations is poorly understood. Antibiotic-induced alterations in the microbiota are known to reduce colonization resistance, increasing susceptibility to specific opportunistic (entero-) pathogens [18,19], such as *Clostridium difficile* and Salmonella [20]. Conversely, in LMICs, mass administration of azithromycin has been shown to significantly reduce childhood mortality, possibly through a reduction in enteropathogenic burden [12,13,21,22]. Antibiotic-driven alterations of early microbiome colonization in high-income settings have been associated with risks of multiple childhood diseases later in life, including obesity [23–27], asthma [28], and diabetes [29–31]. Studies in LMIC children have primarily demonstrated antibiotics’ growth-promoting capacity [32], with lack of reports on the long-term health effects of antibiotic microbiome modulation in these regions.

Foremost among the risks of antibiotics in LMICs is their potential to promote AMR through mutation and selection of bacteria with resistance to antibiotics while increasing the abundance of bacterial AMR genes (ARGs) (termed the resistome) [33]. Mobile genetic elements carrying ARGs can be spread horizontally to bystander bacteria which then acquire the ability to resist antibiotic treatment [34,35]. Antibiotics likely differ in their ability to drive the selection, spread, and persistence of AMR genes in the microbiome and in the degree to which they place children in LMIC communities at risk of infection from pathogens with AMR. Baseline differences in gut microbiome diversity and composition by geographical region and ethnicity may also modulate antibiotic effects [36]. There is paucity of information on the effects of antibiotic use on either the developing child microbiome or resistance gene carriage in LMIC children where antibiotic misuse is on the rise. This systematic review aims to address this knowledge gap by reviewing the literature on the impact of antibiotic exposures on both the faecal bacterial microbiome and resistome composition in children below the age of 2 from LMICs.

## Methods

The Preferred Reporting Items for Systematic Reviews and Meta-analyses (PRISMA) checklist and guidelines were used in the design of the review and the protocol was registered with the International prospective register for systematic reviews (PROSPERO), with ID CRD42022307691.

### Search strategy

The search strategy was designed to include all human studies in infants below the age of 2 from LMICs with a documented use of antibiotics and profiling of the gut microbiome composition and/or resistome following antibiotic usage. Study inclusion criteria included the following study types: case-control studies, cohort studies, randomized control trials (RCTs), and clinical trials. Studies had to characterize the bacterial microbiome using molecular techniques and AMR at the genotypic level. Exclusion criteria included studies with antibiotic resistance measured using phenotypic methods and studies performed in animals and studies that looked at microbiomes other than the gastrointestinal microbiome.

Five electronic databases, MEDLINE (1946 to 28 January 2023), EMBASE (1947 to 28 January 2023), SCOPUS (1945 to 29 January 2023), WHO Global Index Medicus (searched up to 29 January 2023), and SciELO (searched up to 29 January 2023) were used to conduct the literature searches using the Ovid interface for MEDLINE and EMBASE (see S1 Table). The search terms used included the following keywords: (developing country OR low-income country OR middle-income country) AND (infan* OR child*) AND (antibiotic agent OR anti-bacterial* or antibiotic* or anti-biotic* or antibacterial* OR antimicrob* OR anti-microb*) (gut microbiome OR gastrointestinal microbiome OR microbiota) AND (antibiotic resistome OR metagenome OR metagenomics OR resistom*).

A secondary search strategy identified studies performed in high-income countries (HICs) that also met the above search criteria. A recent review published in 2021 evaluated antimicrobial impacts on the microbiome and dysbiosis [37]. We used this literature search with our more stringent inclusion criteria (specifically, articles that report the antibiotics used in the assessment of the microbiome diversity, studies that use either the metagenomic or 16S RNA approach). We also used the above search strategy for studies published from 2021 to date, minus the LMICs search terms (S2 Table).

### Data extraction and quality assessment

Study screening was performed by 2 independent assessors and a third assessor was used to resolve differences in selected studies. The screening was done in Rayyan [38], a free semiautomated web application, first by title and abstract, then by full text. Thereafter, articles that made the final inclusion list were extracted and summarized. The table was created by modifying the Cochrane data collection form for RCTs. The primary author carried out the data extraction independently and extracted the following items: study design employed, the type and/or class of antibiotics used, length of follow up, and the outcomes assessed: microbiome composition, resistome profiles, or a combination of both.

Quality assessment for each included study was performed independently by 2 researchers (CCL and VH) using the revised Cochrane risk-of-bias 2 (RoB 2) tool for randomized studies that is a standardized assessment tool to grade study biases at different stages of a clinical trial based on empirical and theoretical evidence [39]. In the event of disagreements in quality grading between the 2 assessors, a third independent assessor was used to resolve the disagreements. See S3 Table for the PRISMA checklist.

### Outcome measures

The primary outcome measures were differences in mean microbiome diversity and composition following antibiotic usage in an antibiotic treatment group compared to placebo. The outcomes were assessed using either 16S ribosomal ribonucleic acid (rRNA) and/or metagenomics. The 2 methods profile the microbiome at different sequencing depth and 16S rRNA has been reported to being prone to amplification bias due to the regions chosen as opposed to metagenomics hence observed differences in the taxa identified by either method. The review extracted alpha diversity as measured by richness (number of observed taxa at different taxonomic scales such as operational taxonomic units (OTUs) found in a sample), Chao 1, Shannon and/or Simpson indices. OTUs focus on the richness of observed taxa without regard of the frequencies, the Shannon index measures evenness and richness while the Simpson index is more sensitive to species evenness [40]. Extracted beta diversity included pairwise Bray–Curtis dissimilarities and weighted and unweighted uniFrac distances [41]. Composition between arms was extracted at different taxonomic scales (phylum, family, genus, and species).

Resistome composition was extracted when assessed by comparing total resistance gene abundance at baseline and following treatment as well as between placebo and treatment groups using dissimilarity matrices calculated by the Bray–Curtis [42]. Relative abundance of antibiotic-specific genes following treatment was also extracted.

## Results

**Fig 1:**
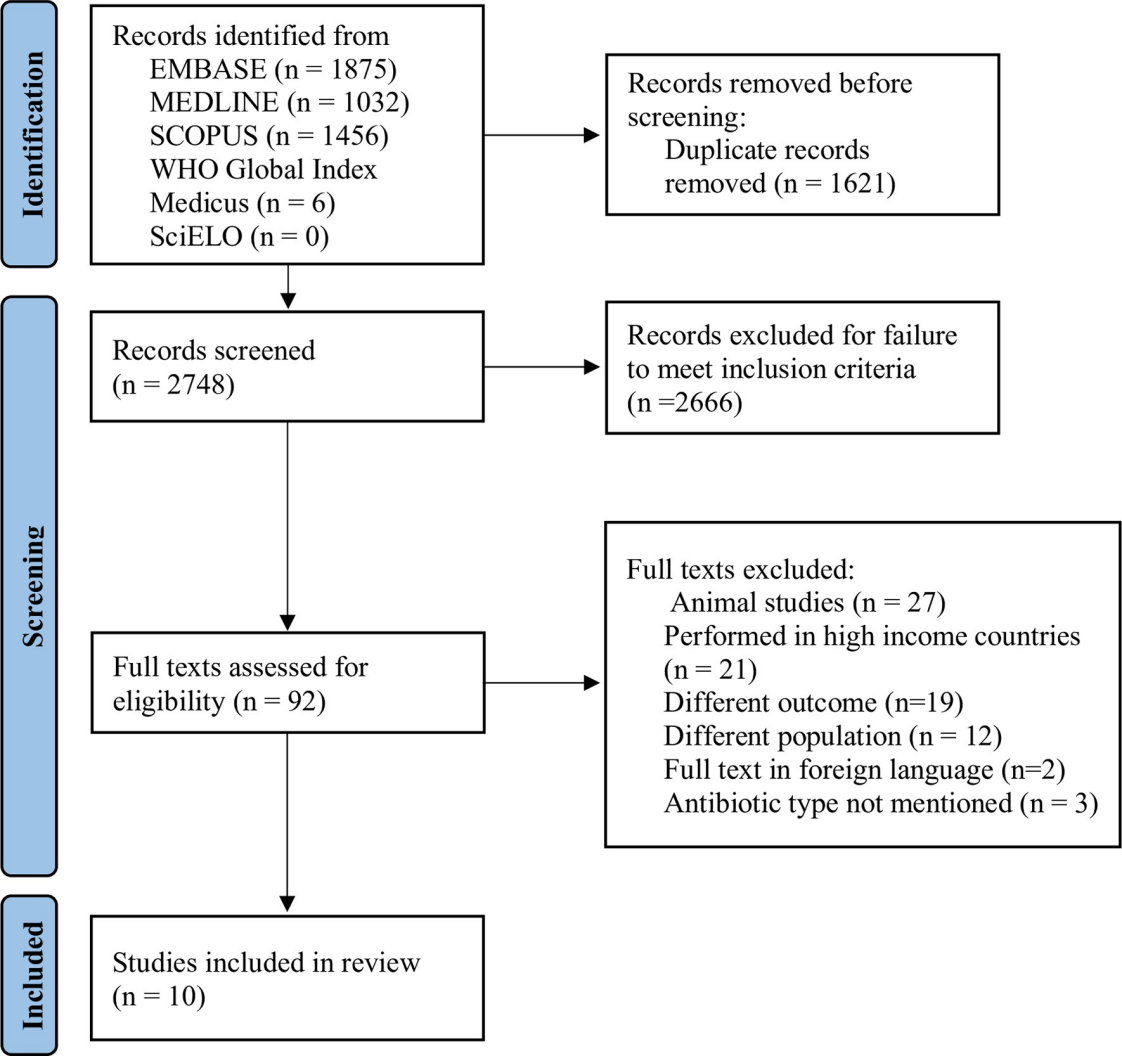
PRISMA flow diagram for the systematic review articles on the influence of infant antibiotic usage on the development of AMR genes and gut microbiome composition. AMR, antimicrobial resistance.

**Fig 2:**
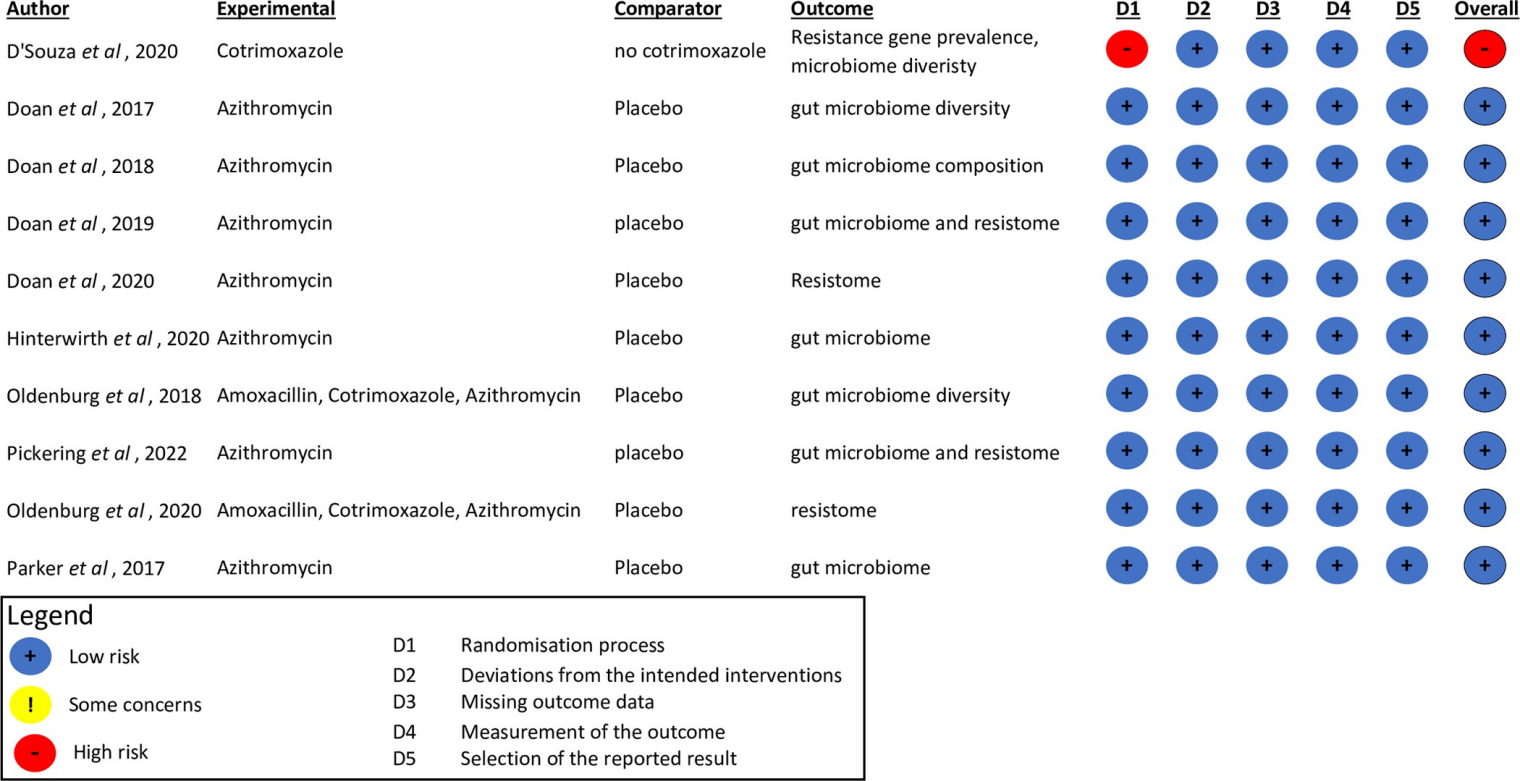
The risk of bias results of the 10 included LMICs RCTs using the Risk of Bias 2 (RoB 2) tool. D’Souza and colleagues (2020) [15], Doan and colleagues (2017) [43], Doan and colleagues (2018) [44], Doan and colleagues (2019) [45], Doan and colleagues (2020) [46], Hinterwirth and colleagues (2020) [47], Oldenburg and colleagues (2018) [48], Pickering and colleagues (2022) [49], Oldenburg and colleagues (2020) [50], Parker and colleagues (2017) [51]. LMIC, low- and middle-income country; RCT, randomized control trial.

The literature search identified 4,369 articles and these were exported to Rayyan. Upon screening of titles and abstracts, 1,621 duplicate records were removed resulting in 2,748 articles, of these 2,666 did not meet the inclusion criteria and were removed (Fig 1). Full-text screening of the remaining 92 studies resulted in exclusion of 82 studies due to 21 being performed in HICs, 3 not mentioning the type of antibiotic the participants took, 27 involving animal experiments, 19 assessing the wrong outcomes, and 12 having the incorrect population group. This resulted in the inclusion of 10 studies. Included studies were assessed for bias, illustrated in Fig 2. All studies had a low risk of bias with the exception of D’Souza and colleagues [15] that had a high risk of bias due to randomization, as the caregivers were not blinded to treatment allocation. Due to the low number of the studies, risk of bias assessment results were not used to exclude studies.

A secondary analysis identified studies evaluating antibiotic impacts on the microbiome and resistome in HIC. The screening and exclusions identified 2 studies eligible for inclusion (see S1 Fig). Significant heterogeneity in infant health status, study design, antibiotic indication, and antibiotic class between studies identified in HIC versus LMIC precluded a comparison between HIC and LMIC studies. Data on these studies and their results are described in S1 Text, S2 Table, and S2 and S3 Figs.

### Characteristics of the included studies

Ten studies from LMIC were included in the final analysis, and all ten were RCTs. Characteristics of the studies are outlined in Table 1. The studies represented diverse regions, 1 study was carried out in South Africa [15], 4 in Niger [43–46], 3 in Burkina Faso [47,48,50], 1 in India [51], and 1 in Malawi [49]. Two of the studies from Burkina Faso [48,50] were published separately but assessed the same patient population. Hence, for this review, the study population from these 2 studies will be referred to as one. The studies assessed a limited number of antibiotics, including cotrimoxazole (CTX, 3 studies), azithromycin (AZI, 9 studies), and amoxicillin (AMX, 2 studies), with 1 study assessing all 3 antibiotics.

**Table 1:** Summary characteristics of the included studies.

Author	Country	Age (months)	Antibiotic, dose, frequency of administration	Genomic methodology	Sampling time point and storage temp^+^	Key results			Comparator (*n*)	Study type	Sample size
						Microbiome		Resistome			
						Diversity	Composition				
[15]	South Africa	0–12 *	CTX, <5 kg: 20 mg trimethoprim/100 mg sulfamethoxazole orally or 5–15 kg: 40 mg trimethoprim and 200 mg sulfamethoxazole once daily for 6 months	Metagenomic sequencing	Day 0, 4 months, 6 months −80°C	No significant differences in α-diversity between treatment arm and placebo	No significant difference in microbial taxa between arms	Significant increase in resistance gene α-diversity and prevalence at 4 and 6 months in treatment arm	No CTX treatment (29)	RCT	63
[49]	Malawi	1–59	AZI, 20 mg/kg Single dose at baseline, 6, 12, and 18 months	Metagenomic sequencing	Day 0 and 24 months −80°C	No significant differences in α-diversity between treatment arm and placebo	No significant difference in composition of taxa between arms	Significant increase in macrolide resistance genes following treatment	Placebo (61)	RCT	122
[43]	Niger	1–60	AZI 20 mg/kg Single dose at baseline	16S rRNA gene sequencing	Day 0 and day 5 −80°C	Significant reduction in α-diversity in treatment arm compared to placebo	Significant differences in composition of taxa between arms. enrichment of gram-positive anaerobes like *Blautia* and decrease in *Bacteroidetes*	n/a	Placebo (40)	RCT	80
[44]	Niger	1–60	AZI 20 mg/kg Single dose at baseline and 6 months	Metagenomic sequencing	Day 0 and 12 months −80°C	Significant reduction in α-diversity in treatment arm compared to placebo	Significant differences in composition between arms	n/a	Placebo (150)	RCT	300
[45]	Niger	1–60	AZI 20 mg/ kg Single dose at baseline, 6, 12, and 18 months	Metagenomic sequencing	Day 0 and 24 months −80°C	No significant reduction in richness in treatment compared to placebo arm	No significant changes in composition between arms	Significant increase in macrolide resistance genes in treatment arm	Placebo (150)	RCT	300
[46]	Niger	1–59	AZI 20 mg/kg Single dose at baseline, 6, 12, 18, 24, 40, 36, and 42 months	Metagenomic sequencing	Day 0, 36 months, and 48 months −80°C	n/a	n/a	Significantly higher macrolide resistance genes in AZI arm	Placebo (555)	RCT	1,073
[47]	Burkina Faso	6–59	AZI 10 mg/kg dose on day 1 followed by 5 mg/kg daily for 4 days 5-day course at baseline	Metagenomic sequencing.	Day 0 and day 5 −80°C	n/a	Significant differences in abundance of 10 genera including *Campylobacter* spp. in AZI arm	n/a	Placebo (30)	RCT	61
[48]**	Burkina Faso	6–59	AZI, 10 mg/kg dose on day 1 and then 5 mg/kg once daily for 4 days AMX 25 mg/kg/d twice daily doses or CTX 240 mg once daily 5-day course at baseline	16S rRNA gene sequencing	Day 0 and day 5 −80°C	Significant differences in α-diversity across the treatment arms with diversity lowest in AZI arm and highest in placebo arm		n/a	Placebo (29)	RCT	115
[50]**				Metagenomic sequencing	Day 0 and day 5 −80°C	n/a	n/a	Significant increase in macrolide resistance genes in azithromycin arm. Higher sulfonamide resistance genes in all 3 antibiotics arms	Placebo (29)	RCT	115
[51]	India	6–11	AZI 20 mg/kg 3-day course at baseline	16S rRNA gene sequencing	Day 0 and day 14 No information	Reduced richness in treatment arms compared to placebo	Reduced relative abundance of *Proteobacteria* and *Verrucomicrobia* in AZI arm	n/a	Placebo (58)	RCT	114

*These participants were HIV-exposed but uninfected.

** The 2 RCTs that reported the use of 3 antibiotics assessed the same population and each participant was randomized to receive only 1 antibiotic.

^+^ Refers to long-term sample storage temperature before analysis.

AMX, amoxicillin; AZI, azithromycin; CTX, cotrimoxazole; n/a, not applicable; RCT, randomized controlled trial; rRNA, ribosomal ribonucleic acid.

Gut microbiota analyses were performed using 16S rRNA sequencing (3 studies) and metagenomic shotgun sequencing (7 studies), and 5 studies evaluated the presence of resistance genes using metagenomic sequencing. The comparator for all studies, excluding one, was a placebo which was an oral suspension of matching color and taste. One study from South Africa [15] did not use a placebo (Table 1). The difference in AZI dosing, with the widely reported dose being 20 mg/kg, did not seem to contribute on the observed effects of the antibiotic on the gut microbiome. CTX doses, reported at 20 mg trimethoprim/100 mg sulfamethoxazole of <5 kg or 40 mg trimethoprim/200 mg sulfamethoxazole for 5 to 15 kg [15,48], did not result in different impacts as both studies reported nonsignificant effects of the antibiotic on the microbiome.

Meta-analysis of these studies was considered. However, preliminary analysis of only the AZI studies showed unacceptably high heterogeneity precluding a legitimate meta-analysis (S4 Fig, I^2^ = 84.83%). This heterogeneity could be caused by the considerable differences in antibiotic class, sampling strategy, analysis workflows, diversity indices (such as Shannon, Simpsons, and OTU count), reported taxonomic levels, and sequencing methods (16S and metagenomics) used to characterize the gut microbiome and the resistome between the studies.

### Impact of antibiotic use on the gut microbiome diversity

**Fig 3:**
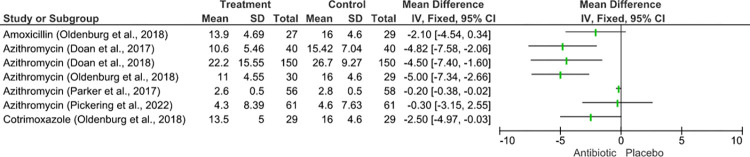
Forest plot indicating the effects of antibiotic treatment on alpha diversity as measured by the Shannon’s index. Doan and colleagues (2017) [43], Doan and colleagues (2018) [44], Oldenburg and colleagues (2018) [48], Pickering and colleagues (2022) [49], Parker and colleagues (2017) [51].

**Fig 4:**
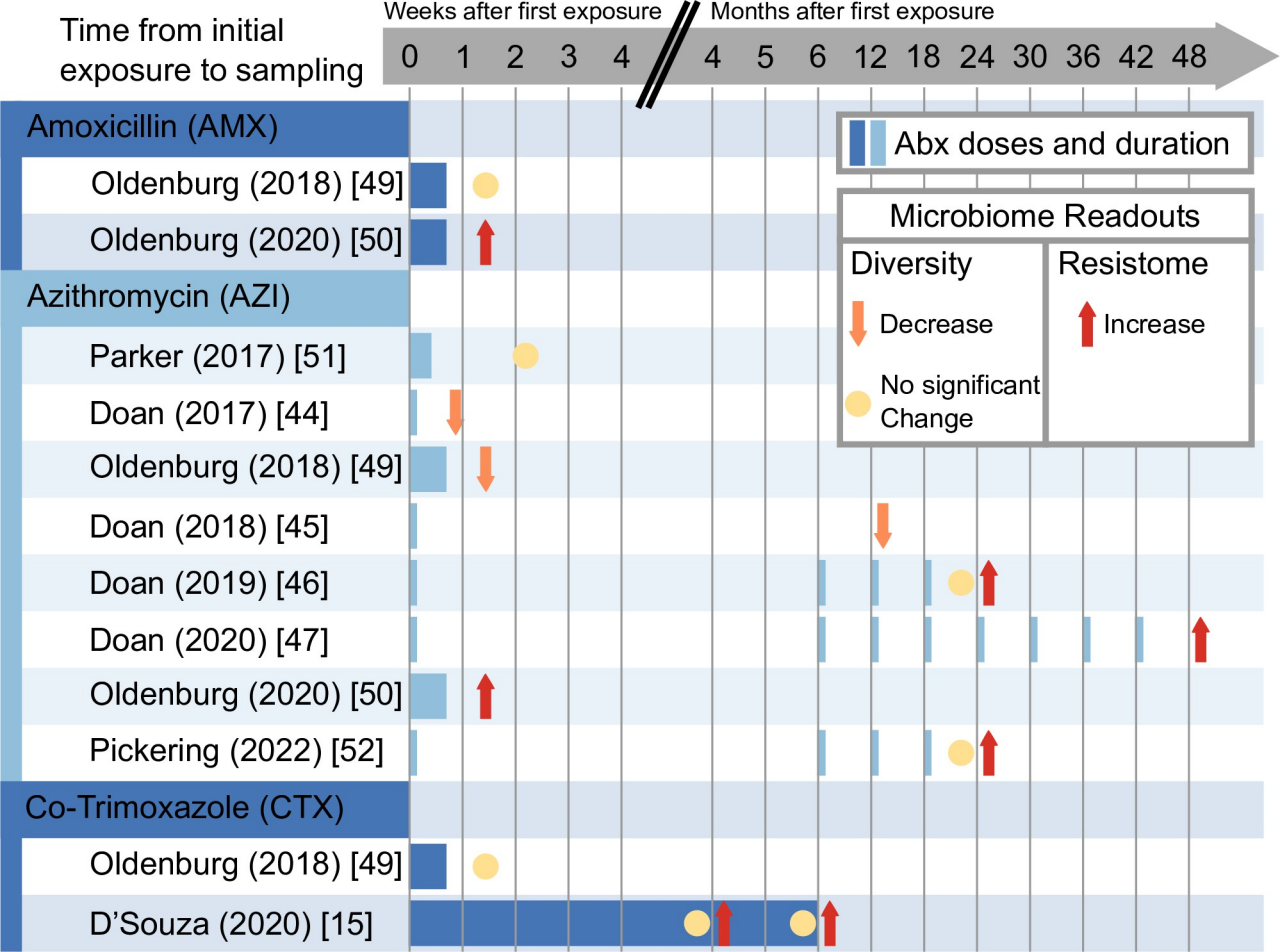
Effects of antibiotic use on alpha diversity and resistome abundance in relation to class of antibiotic, antibiotic duration, and sampling time. D’Souza (2020) [15], Doan (2017) [43], Doan (2018) [44], Doan (2019) [45], Doan (2020) [46], Hinterwirth (2020) [47], Oldenburg (2018) [48], Pickering (2022) [49], Oldenburg (2020) [50]. CTX, cotrimoxazole, AZI, azithromycin, AMX, amoxicillin. Reduction in microbiome diversity, (orange arrow pointing up). No significant change in microbiome diversity, (yellow circle). Increase in resistome abundance, (red arrow pointing up) dosing time point and duration (blue solid lines).

Nine studies reported the effects of antibiotic use on gut microbiome diversity (Figs 3 and 4). Antibiotic impact on α-diversity differed depending on antibiotic type, duration of use, and follow-up time (Fig 4). Different indices were used to measure alpha and beta diversity across the studies, with some studies reporting on either the Shannon index [48,49], which are shown in an overview in Fig 3, or a combination of the Shannon and Simpsons indices, whose overall effects are shown in Fig 4 [43,44,48,51] while taxonomic richness was reported in 2 studies [15,45].

Two studies [45,49] evaluating microbiome composition 6 months after 4 biannual administrations of AZI found no significant changes in richness and α-diversity as measured by Shannon index. Four studies evaluating short courses of CTX, AZI, or AMX, reported a decrease in microbial α-diversity, using the Shannon index and OTU counts, directly 5 days posttreatment [43,44,48] and 12 days posttreatment [51]. Only one study (D’Souza) reported an increase in α-diversity, which was evaluated at 4 and 6 months of daily CTX usage in HIV-exposed but uninfected infants but was not significant [15].

Three studies reported on β-diversity [15,43,49]. One found a decrease in intra-group beta diversity following CTX treatment [15] when compared to the placebo as measured by pairwise Bray–Curtis dissimilarities indices. The dissimilarity was significantly lower in the treatment arms compared to the placebo arms. In contrast, 2 studies [43,49] reported no significant changes in beta diversity between the treatment and placebo groups at 5 days and 6 months following a single course of AZI.

### Changes in microbiome composition following antibiotic administration

Changes in taxonomic composition were assessed at various taxonomic ranks though comparability was limited due to reporting differences between studies (Table 1). Three studies did not report any significant changes in composition following treatment with CTX [15] and AZI [45,49]. *Four studies reported a significant differences in microbiome taxa composition following treatment with AZI* [43,44,47,51]. The most notable changes were reductions in bacteria from the Proteobacteria phylum, namely *Campylobacter hominis*, *Campylobacter jejuni* and *Campylobacter ureolyticus* following a 5-day course of AZI [47]. A study in Malawi reported an increase in 1 putative Proteobacteria enteropathogen (*Escherichia albertii)* following AZI administration [49]. Additionally, AZI treatment resulted in a reduction in *Bacteroides* [43,52] and increase in Firmicutes [51].

### Impact of antibiotics on antimicrobial resistance genes

Five studies assessed ARG abundance following CTX, AZI, and AMX therapy. One study [15], in which CTX was administered daily to HIV-exposed infants for 6 months, reported significantly higher total resistance gene prevalence and diversity in the treatment group as compared to placebo group at 4 and 6 months of treatment. CTX treatment had a significantly lower dissimilarity compared to the placebo when assessed using the Bray–Curtis dissimilarities. Three studies [45,46,49] evaluated the impact of repeated short courses of AZI treatment on long-term resistance gene prevalence. They all reported a significant increase in macrolide resistance gene abundance 6 months after 2 years of biannual AZI administration [45,49] as well as an increase in resistance determinants to several non-macrolide antibiotics, specifically beta-lactams, following 4 years of biannual AZI administration [46]. One study compared 5-day courses of AZI, AMX, and CTX treatment [50] and assessed the prevalence of resistance genes 5 days posttreatment. AZI and CTX treatment significantly increased macrolide and sulfonamide resistance determinants, respectively, compared to placebo. AMX also increased resistance gene richness significantly following treatment (Table 2).

**Table 2:** Effects of antibiotics on the AMR gene abundance.

Antibiotic (Class)	Time from exposure to analysis	Findings	References
CTX (sulfonamide)	Antibiotic exposure was throughout the 6 months period, analysis at day 0, month 4, and month 6	**Significant increase** in total resistance gene α-diversity from baseline to 4 months *p* = 0.035 and baseline to 6 months *p* = 0.046.	[15]
	Five days of antibiotic exposure, analysis 5 days from treatment cessation	**Significant increase in sulfonamide and trimethoprim resistance** genes in treatment arm. Cotrimoxazole samples were more than **3 times likely to have trimethoprim resistance** (RR, 3.29; 95% CI, 1.08–9.95; *p* = 0.04) compared to placebo.	[50]
AZI (macrolide)	One day of antibiotic exposure every 6 months, analysis 6 months after the fourth dose	**Significant increase in macrolide resistance** gene expression in treatment arm at 24 months (*p* < 0.001) compared to placebo. Prevalence of macrolide gene expression in treatment arm was 16.7% (95% CI: 9.3–24.7).	[45]
	One day of antibiotic exposure every 6 months, analysis 6 months after the fourth dose	**Significant increase in macrolide resistance** genes in treatment arm at 24 months (*p* < 0.001 ) compared to placebo.	[49]
	One day of antibiotic exposure every 6 months, analysis 6 months after the eighth dose	**Significant increase in macrolide resistance** genes in treatment arm at 48 months. **Macrolide resistance determinants 7.5 times higher in treatment arm compared to placebo** (95% CI, 3.8–23.1) at 48 months. **Increases in multiple non-macrolide resistance genes at 36 months**, including beta-lactam resistance determinants by 2.1 times (95% CI, 1.3–4.0).	[46]
	Five days of antibiotic exposure, analysis 5 days from treatment cessation	**Significant increase in macrolide and sulfonamide resistance** genes in treatment arm. Treatment arm **had twice as much macrolide resistance** (RR, 2.61; 95% CI 1.55–4.42; *p* < 0.001) compared to the placebo.	[50]
AMX (beta-lactam)	Five days of antibiotic exposure, analysis 5 days from treatment cessation	**Significant increase in sulfonamide resistance** genes in treatment arm. (RR 15.3, 95% CI 1.80–129.1; *p* < 0.001) Mean resistance gene **richness was significantly higher** in treatment arm compared to placebo (42.6 vs. 23.9, *p* = 0.02).	[50]

CTX, Cotrimoxazole, AZI, Azithromycin, AMX, Amoxicillin, RR, risk ratio, CI, Confidence interval.

### Antibiotic class-specific impacts on microbiome and resistome composition

To understand if impact on microbiome diversity and composition differed by antibiotic class in infants from LMIC, we assessed the differences in alpha diversity, composition, and resistome between treatment and control arms by specific antibiotic class.

#### Azithromycin

The majority of included studies from LMIC evaluated the impact of the broad-spectrum macrolide, AZI, on the microbiome. These studies were carried out in Malawi [49], Niger [43–45], Burkina Faso [48,50], and India [51]. Antibiotic dosing schedules and fecal sampling time points differed significantly across the studies. Generally, treatment with AZI reduced alpha diversity of the gut microbiota compared to the placebo arm. Of note was an increase in potential enteropathogens following AZI treatment in children in Malawi [49].

AZI administration increased macrolide resistance gene abundance across all studies, with fecal samples twice as likely to have macrolide resistance determinants 5 days post-antibiotics (compared to the placebo) [50] and macrolide resistance remaining significantly increased (compared to placebo) as long as 6 months following treatment [45,49]. Forty-eight months of biannual AZI administration concomitantly increased non-macrolide resistance determinants, including a greater abundance of beta-lactam-resistance determinants, a commonly used antibiotic class in LMIC settings [46].

#### Cotrimoxazole

CTX, a sulfonamide, has broad antimicrobial activity targeting both gram-negative and gram-positive microorganisms. Three highly heterogenous studies evaluated CTX: 1 in South Africa [15] and 2 in Burkina Faso that used the same study population to assess the impacts of CTX on gut microbiome composition [48] and resistome [50]. Study populations differed significantly, with HIV-exposed children studied in South Africa and healthy infants in Burkina Faso. Antibiotic exposure was also different, with daily administration for 6 months in South Africa versus one 5-day course in Burkina Faso. Sampling time frame also contrasted between the 2 studies, with the South African study conducting analyses while the patients were still taking the medication at 4 and 6 months and the Burkina Faso study conducting the analysis 5 days post treatment cessation.

Independent of treatment duration (5 days versus 6 months) and time of sampling (5 days post therapy or during long-term therapy), CTX significantly increased total resistance genes abundance and trimethoprim and sulfonamide-specific resistance genes.

#### Amoxicillin

AMX, a broad spectrum beta-lactam, was investigated in 1 study population in Burkina Faso for its short-term (5 days posttreatment) impact on the microbiome [48] and gut resistome following a twice daily 5-day treatment course [50]. AMX did not have a significant impact on the microbiome composition compared to the placebo. In the short term, the same 5-day course significantly increased mean resistance gene richness compared to placebo and beta-lactam resistance was highest in the AMX group compared to the CTX and AZI antibiotics arms, albeit nonsignificantly.

## Discussion

To our knowledge, this systematic review, which synthesizes the impacts of antibiotic use on both gut microbiome composition and resistance genes in infants, is the first to focus on infants in LMIC settings. The review found that antibiotics variably impacted microbiome diversity and composition, with effects likely mediated by antibiotic class, duration of administration, and follow-up time. Multiple studies included in the review also showed that antibiotic administration, namely AZI, can shift taxonomic composition of the microbiome, particularly by reducing enteropathogen burden. Finally, antibiotic administration expands both the total and antibiotic class-specific resistome. Even short antibiotic exposures are able to increase AMR genes for months. The magnitude and persistence of these change is likely dictated by duration of antibiotic exposure and class.

The range of antibiotics in the included studies was limited, assessing only AZI, CTX, and AMX (1 study), with AZI being the most widely reported antibiotic. While AZI is not the most commonly used antibiotic in the treatment of childhood infectious syndromes, its capacity to reduce childhood mortality through prophylactic mass administration in high-risk pediatric populations [45], does make it relevant for study in LMIC. No studies conducted in LMICs were found evaluating some of the most commonly used antibiotics of childhood with capacity for anaerobic depletion such as clindamycin and metronidazole.

Antibiotic duration was also found to be a key determinant of the impact on microbiome structure. Even short courses of antibiotics like AZI were able to immediately reduce microbiota diversity [51] with persistent reductions in richness of the microbiota for up to 6 months following administration [44]. A short course of CTX diminished alpha diversity indexes [48], whereas chronic use increased diversity indexes over time [15]. AZI demonstrated a consistent and persistent alteration in taxonomic composition in line with experimental data in adults [53,54].

The review also showed that antibiotic administration can alter the taxonomic composition of the microbiome in LMIC children. A key question is whether antibiotics reduce overall pathogen burden and risk of infections or render children more susceptible to infections—possibly through depletion of the commensal microbiota and loss of colonization resistance. Mouse studies have suggested that early life exposures to antibiotics increase susceptibility to bacterial enteropathogens in adulthood [55]. In the few pediatric studies where enteropathogens were considered, antibiotics tended to reduce enteropathogenic burden in the microbiome. Targeted enteropathoge AZI administration reduced *Campylobacter* spp. relative abundance in Niger and Burkina Faso [45,47] and reduced Proteobacteria with pathogenic potential in India [51]. One study showed an increase in the relative abundance of 1 putative enteropathogen (*E*. *albertii)* in Malawi [49]. These results suggest that 16S and metagenomic analyses may be insufficient to identify clinically-relevant enteropathogens, and complementary targeted enteropathogen testing may be required to characterize antibiotic impacts on enteropathogen burden. In contrast, antibiotic studies in adults have shown increases in enteropathogens post-antibiotics, primarily *C*. *difficile* [56] and vancomycin-resistant enterococci [57], underscoring the complexity of these positive and negative associations with antimicrobial use across age spectra.

Finally, our systematic review found that antibiotics drove total and antibiotic class-specific increases in AMR gene determinants in all included studies. A second key question is to what degree antibiotic usage places a child at risk for infections with antimicrobial resistant pathogens, with AMR gene carriage being a surrogate endpoint in this pathway. AZI consistently increased macrolide resistance that persisted up to 6 months following administration. Longer biannual use notably also increased resistance determinants to non-macrolide antibiotics. In parallel, CTX increased total resistance gene diversity, sulfonamide and trimethoprim resistance determinates. Children in LMICs are subject to multiple rounds of appropriate and inappropriate antibiotics, and therefore, these studies are likely a gross underestimation of the impact of antibiotics across a child’s early life [11,58].

A remaining question, that this systematic review did not address, is how antibiotic-induced reductions in diversity and disruption of the microbiome may impact the biologic functions of the infant microbiome and how these alterations translate into health and illness outcomes for infants in LMICs. The microbiome plays multiple roles in hosts, including immune maturation, immune regulation, and host metabolism [59]. Antibiotic-induced losses in diversity may result in reduction of organisms that play key roles in these biologic functions, potentially altering infants’ immunity to exogenous pathogens, alongside nutrition and growth outcomes. Mouse studies have shown that antibiotic administration in early life increases subsequent risk of atopic disease—such as asthma and adiposity [60,61]. Similarly, unknown is how alterations in AMR gene abundance translate into a child’s short- and long-term risk of infection from an AMR-bearing pathogen. Although metagenomic sequencing can identify changes in AMR genes, it is difficult to deduce from which bacteria those genes are derived. A key limitation of genomic resistome analyses is therefore bacterial host assignment and whether increases in genomic AMR genes translate to clinically relevant phenotypic resistance. Research is urgently needed to understand how antibiotics may impact the functional microbiome diversity [62], phenotypic resistance in enteropathogens, and risk of disease, in order to better inform antibiotic prescribing practices and health policy around AMR and population-level antibiotic administration.

Limitations of this systematic review were the paucity of studies identified and included, the limited number of countries studies were performed in, and the lack of diversity in antibiotic classes. The available data was dominated by 2 large clinical trials in Africa, MORDOR [63] and ARMCA [64]. Other included studies were highly heterogeneous with differing antibiotic dosing schemes and testing time points even within antibiotic class. Thus, both the generalizability and comparability of results is constrained and precluded pooling data and permitting a meta-analysis. While most individual studies detected significant impacts of antimicrobial usage on microbiome composition and resistome, a few studies did not report any significant treatment effects [15,45,49]. This may have been due to a true lack of effect, timing of sampling, or insufficient sample size. Lastly, few studies included long-term sampling, limiting the understanding of how long antibiotic-induced alterations in the microbiome and resistome persist and the resilience of the pediatric LMIC microbiome following antibiotic challenge.

Notable strength of this review is that it provides a comprehensive overview and needed synthesis of the available data on antibiotic usage and microbiome in pediatric LMIC populations with high incidences of antibiotic use. This review thereby maps the current state of knowledge, highlighting available data alongside persistent research gaps that can guide both policy and research agendas. The included studies clearly show that antibiotic administration results in large-scale alterations of the microbiome and resistome with enough evidence to alert policy makers to the adverse and indirect impacts of (mass) antibiotic administration. In parallel, the review highlights key future research needs, namely moving from how alterations in the microbiome and resistome translate to tangible health benefits or risks for pediatric populations, including risk of bacterial infections with AMR.

Our systematic review also provides a mapping to move this field forward. It can be used to address the urgent need for a harmonized methodology to permit comparison of the impacts of antibiotic administration across antibiotic class, geographic setting, and time. Future studies should focus on pediatric populations in LMIC and evaluate commonly used pediatric antibiotics, incorporating sampling at the start, during, and following antibiotic administration, including long-term follow-up. Studies should be adequately powered and strive to employ metagenomic sequencing techniques with sufficient sequencing depth to describe genus and species level antibiotic perturbations alongside alterations in the resistome and functional metagenome. Harmonized annotation pipelines and uniform outcome measures will permit comparability across studies. When possible, sequencing should be combined with bacterial culture to permit host annotation for key resistance genes and host enteropathogens. Open-source data repositories will permit researchers to then re-analyze data across studies, permitting policy makers and clinicians to draw actionable conclusions from this research.

In summary, antibiotics in children in LMIC disrupt the diversity and taxonomy of the gut microbiome and drive long-term expansion of ARG determinants. There is a paucity of research describing the impacts of class-specific antibiotics on microbiome diversity and ARG development in infants in LMIC. Given the vulnerability of children in LMIC to infections, the frequency of antibiotic consumption in their early lives, and the growing global threat of infections with AMR, harmonized research initiatives are urgently needed to understand how antibiotic perturbations on the microbiome translate into health benefits or risk of disease.

## Supporting information

S1 PRISMA Checklist PRISMA 2020 Checklist. (DOCX)

S1 Fig PRISMA flow diagram. Results of searches from 2021 to August 2022 in high-income countries.
(TIF)

S2 Fig Forest plot indicating the effects of antibiotic treatment on alpha diversity as measured by the Shannon’s index in low- and middle-income countries (upper graph) and high-income countries (lower graph). Multiple* refers to penicillin + gentamicin, amoxicillin+clavulanic acid + gentamicin, or amoxicillin + cefotaxime. Doan and colleagues (2017) [43], Doan and colleagues (2018) [44], Oldenburg and colleagues (2018) [48], Pickering and colleagues (2022) [49], Parker and colleagues (2017) [51], Kwon and colleagues (2020) [65], Wei and colleagues (2018) [66], Bai and colleagues (2017) [67], Reyman and colleagues (2022) [68].
(TIF)

S3 Fig Estimates indicating the effects of antibiotic treatment on alpha diversity as measured by the Simpson’s index in low- and middle-income countries (upper graph) and high-income countries (lower graph). Multiple* refers to penicillin + gentamicin, co-amoxiclav + gentamicin or amoxicillin + cefotaxime. Doan and colleagues (2017) [43], Doan and colleagues (2018) [44], Oldenburg and colleagues (2018) [48], Kwon and colleagues (2020) [65], Bai and colleagues (2017) [67].
(TIF)

S4 Fig Pool estimates of the effect of azithromycin on mean alpha diversity as measured by Shannon’s index. Estimates were obtained using random effects restricted maximum likelihood model. Doan and colleagues (2017) [43], Doan and colleagues (2018) [44], Oldenburg and colleagues (2018) [48], Pickering and colleagues (2022) [49], Parker and colleagues (2017) [51].
(TIF)

S1 Table Lists of databases searched and results obtained from each dataset for low- and middle-income countries. (XLSX)

S2 Table Lists of databases searched and results obtained from each dataset for the low- and middle-income countries vs. high-income countries comparisons. (XLSX)

S3 Table Extended PRISMA checklist. (XLSX)

S1 Text Low- and middle-income countries vs. high-income countries comparisons, systematic review results and discussion. (DOCX)

## Abbreviations

AMR: antimicrobial resistance
AMX: amoxicillin
AZI: azithromycin
CTX: cotrimoxazole
HIC: high-income country
LMIC: low- and middle-income country
OTU: operational taxonomic unit
RCT: randomized control trial
rRNA: ribosomal ribonucleic acid
WHO: World Health Organization

## References

[1] Jonas O, Irwin A, Berthe FC, Le Gall F, Marquez PV. Drug-resistant infections: a threat to our economic future (Vol. 2): final report (English). HNP/Agriculture Global Antimicrobial Resistance Initiative, Washington, D.C.: World Bank Group; 2017. Available from: http://documents.worldbank.org/curated/en/323311493396993758/final-report.

[2] O’Neill J. Review on Antimicrobial Resistance Antimicrobial Resistance: Tackling a crisis for the health and wealth of nations. Rev Antimicrob Resist. 2014. Available from: https://amr-review.org/sites/default/files/AMR%20Review%20Paper%20-%20Tackling%20a%20crisis%20for%20the%20health%20and%20wealth%20of%20nations_1.pdf.

[3] O’Neill J. Tackling drug-resistant infections globally: final report and recommendations. Government of the United Kingdom; 2016 May. Available from: https://apo.org.au/node/63983.

[4] Murray CJ, Ikuta KS, Sharara F, Swetschinski L, Aguilar GR, Gray A, et al. Global burden of bacterial antimicrobial resistance in 2019: a systematic analysis. Lancet. 2022;399:629–655. doi: 10.1016/S0140-6736(21)02724-0 35065702 PMC8841637

[5] World Health Organisation. Antimicrobial resistance. 2021 [cited 2022 Jul 14]. Available from: https://www.who.int/news-room/fact-sheets/detail/antimicrobial-resistance.

[6] Bell BG, Schellevis F, Stobberingh E, Goossens H, Pringle M. A systematic review and meta-analysis of the effects of antibiotic consumption on antibiotic resistance. BMC Infect Dis. 2014;14:13. doi: 10.1186/1471-2334-14-13 24405683 PMC3897982

[7] Goossens H, Ferech M, Vander Stichele R, Elseviers M, ESAC Project Group. Outpatient antibiotic use in Europe and association with resistance: a cross-national database study. Lancet Lond Engl. 2005;365:579–587. doi: 10.1016/S0140-6736(05)17907-0 15708101

[8] Olesen SW, Barnett ML, MacFadden DR, Brownstein JS, Hernández-Díaz S, Lipsitch M, et al. The distribution of antibiotic use and its association with antibiotic resistance. Ferguson NM, Jha P, editors. eLife. 2018;7:e39435. doi: 10.7554/eLife.39435 30560781 PMC6307856

[9] Browne AJ, Chipeta MG, Haines-Woodhouse G, Kumaran EPA, Hamadani BHK, Zaraa S, et al. Global antibiotic consumption and usage in humans, 2000–18: a spatial modelling study. Lancet Planet Health. 2021;5:e893–e904. doi: 10.1016/S2542-5196(21)00280-1 34774223 PMC8654683

[10] Tadesse BT, Ashley EA, Ongarello S, Havumaki J, Wijegoonewardena M, González IJ, et al. Antimicrobial resistance in Africa: a systematic review. BMC Infect Dis. 2017:17. doi: 10.1186/s12879-017-2713-1 28893183 PMC5594539

[11] Williams PCM, Isaacs D, Berkley JA. Antimicrobial resistance among children in sub-Saharan Africa. Lancet Infect Dis. 2018;18:e33–e44. doi: 10.1016/S1473-3099(17)30467-X 29033034 PMC5805911

[12] Keenan JD, Bailey RL, West SK, Arzika AM, Hart J, Weaver J, et al. Azithromycin to Reduce Childhood Mortality in Sub-Saharan Africa. N Engl J Med. 2018;378:1583–1592. doi: 10.1056/NEJMoa1715474 29694816 PMC5849140

[13] Keenan JD, Arzika AM, Maliki R, Boubacar N, Elh Adamou S, Moussa Ali M, et al. Longer-Term Assessment of Azithromycin for Reducing Childhood Mortality in Africa. N Engl J Med. 2019;380:2207–2214. doi: 10.1056/NEJMoa1817213 31167050 PMC6512890

[14] Fink GD’Acremont V, Leslie HH, Cohen J. Antibiotic exposure among children younger than 5 years in low-income and middle-income countries: a cross-sectional study of nationally representative facility-based and household-based surveys. Lancet Infect Dis. 2020;20:179–187. doi: 10.1016/S1473-3099(19)30572-9 31843383

[15] D’Souza AW, Moodley-Govender E, Berla B, Kelkar T, Wang B, Sun X, et al. Cotrimoxazole Prophylaxis Increases Resistance Gene Prevalence and α-Diversity but Decreases β-Diversity in the Gut Microbiome of Human Immunodeficiency Virus–Exposed, Uninfected Infants. Clin Infect Dis. 2020;71:2858–2868. doi: 10.1093/cid/ciz1186 31832638 PMC7778358

[16] Gasparrini AJ, Crofts TS, Gibson MK, Tarr PI, Warner BB, Dantas G. Antibiotic perturbation of the preterm infant gut microbiome and resistome. Gut Microbes. 2016;7:443–449. doi: 10.1080/19490976.2016.1218584 27472377 PMC5154371

[17] Gasparrini AJ, Wang B, Sun X, Kennedy EA, Hernandez-Leyva A, Ndao IM, et al. Persistent metagenomic signatures of early-life hospitalization and antibiotic treatment in the infant gut microbiota and resistome. Nat Microbiol. 2019;4:2285–2297. doi: 10.1038/s41564-019-0550-2 31501537 PMC6879825

[18] Buffie CG, Pamer EG. Microbiota-mediated colonization resistance against intestinal pathogens. Nat Rev Immunol. 2013;13:790–801. doi: 10.1038/nri3535 24096337 PMC4194195

[19] Sequeira RP, McDonald JAK, Marchesi JR, Clarke TB. Commensal Bacteroidetes protect against Klebsiella pneumoniae colonization and transmission through IL-36 signalling. Nat Microbiol. 2020;5:304–313. doi: 10.1038/s41564-019-0640-1 31907407 PMC7610889

[20] Wu H, Ma Y, Peng X, Qiu W, Kong L, Ren B, et al. Antibiotic-induced dysbiosis of the rat oral and gut microbiota and resistance to Salmonella. Arch Oral Biol. 2020;114:104730. doi: 10.1016/j.archoralbio.2020.104730 32371145

[21] Keenan JD, Ayele B, Gebre T, Zerihun M, Zhou Z, House JI, et al. Childhood mortality in a cohort treated with mass azithromycin for trachoma. Clin Infect Dis Off Publ Infect Dis Soc Am. 2011;52:883–888. doi: 10.1093/cid/cir069 21427395 PMC3106233

[22] Porco TC, Gebre T, Ayele B, House J, Keenan J, Zhou Z, et al. Effect of mass distribution of azithromycin for trachoma control on overall mortality in Ethiopian children: a randomized trial. JAMA. 2009;302:962–968. doi: 10.1001/jama.2009.1266 19724043

[23] Block JP, Bailey LC, Gillman MW, Lunsford D, Daley MF, Eneli I, et al. Early Antibiotic Exposure and Weight Outcomes in Young Children. Pediatrics. 2018;142:e20180290. doi: 10.1542/peds.2018-0290 30381474 PMC6317759

[24] Kelly D, Kelly A, O’Dowd T, Hayes CB. Antibiotic use in early childhood and risk of obesity: longitudinal analysis of a national cohort. World J Pediatr. 2019;15:390–397. doi: 10.1007/s12519-018-00223-1 30635840

[25] Miller SA, Wu RKS, Oremus M. The association between antibiotic use in infancy and childhood overweight or obesity: a systematic review and meta-analysis. Obes Rev Off J Int Assoc Study Obes. 2018;19:1463–1475. doi: 10.1111/obr.12717 30035851

[26] Rogawski ET, Westreich DJ, Adair LS, Becker-Dreps S, Sandler RS, Sarkar R, et al. Early Life Antibiotic Exposure Is Not Associated with Growth in Young Children of Vellore, India. J Pediatr. 2015;167:1096–102.e3. doi: 10.1016/j.jpeds.2015.08.015 26372535 PMC5030490

[27] Scott FI, Horton DB, Mamtani R, Haynes K, Goldberg DS, Lee DY, et al. Administration of Antibiotics to Children Before Age 2 Years Increases Risk for Childhood Obesity. Gastroenterology. 2016;151:120–129.e5. doi: 10.1053/j.gastro.2016.03.006 27003602 PMC4924569

[28] Marra F, Marra CA, Richardson K, Lynd LD, Kozyrskyj A, Patrick DM, et al. Antibiotic use in children is associated with increased risk of asthma. Pediatrics. 2009;123:1003–1010. doi: 10.1542/peds.2008-1146 19255032

[29] Chen RY, Mostafa I, Hibberd MC, Das S, Mahfuz M, Naila NN, et al. A Microbiota-Directed Food Intervention for Undernourished Children. N Engl J Med. 2021;384:1517–1528. doi: 10.1056/NEJMoa2023294 33826814 PMC7993600

[30] Clausen TD, Bergholt T, Bouaziz O, Arpi M, Eriksson F, Rasmussen S, et al. Broad-Spectrum Antibiotic Treatment and Subsequent Childhood Type 1 Diabetes: A Nationwide Danish Cohort Study. PLoS ONE. 2016;11:e0161654. doi: 10.1371/journal.pone.0161654 27560963 PMC4999141

[31] Robertson RC, Manges AR, Finlay BB, Prendergast AJ. The Human Microbiome and Child Growth–First 1000 Days and Beyond. Trends Microbiol. 2019;27:131–147. doi: 10.1016/j.tim.2018.09.008 30529020

[32] Gough EK, Moodie EEM, Prendergast AJ, Johnson SMA, Humphrey JH, Stoltzfus RJ, et al. The impact of antibiotics on growth in children in low and middle income countries: systematic review and meta-analysis of randomised controlled trials. BMJ. 2014;348:g2267. doi: 10.1136/bmj.g2267 24735883 PMC3988318

[33] Wright GD. The antibiotic resistome: the nexus of chemical and genetic diversity. Nat Rev Microbiol. 2007;5:175–186. doi: 10.1038/nrmicro1614 17277795

[34] Bello-López JM, Cabrero-Martínez OA, Ibáñez-Cervantes G, Hernández-Cortez C, Pelcastre-Rodríguez LI, Gonzalez-Avila LU, et al. Horizontal Gene Transfer and Its Association with Antibiotic Resistance in the Genus Aeromonas spp. Microorganisms. 2019;7:363. doi: 10.3390/microorganisms7090363 31540466 PMC6780555

[35] Mathers AJ, Peirano G, Pitout JDD. The Role of Epidemic Resistance Plasmids and International High-Risk Clones in the Spread of Multidrug-Resistant Enterobacteriaceae. Clin Microbiol Rev. 2015;28:565–591. doi: 10.1128/CMR.00116-14 25926236 PMC4405625

[36] Gupta VK, Paul S, Dutta C. Geography, Ethnicity or Subsistence-Specific Variations in Human Microbiome Composition and Diversity. Front Microbiol. 2017;8. doi: 10.3389/fmicb.2017.01162 28690602 PMC5481955

[37] McDonnell L, Gilkes A, Ashworth M, Rowland V, Harries TH, Armstrong D, et al. Association between antibiotics and gut microbiome dysbiosis in children: systematic review and meta-analysis. Gut Microbes. 2021;13:1–18. doi: 10.1080/19490976.2020.1870402 33651651 PMC7928022

[38] Ouzzani M, Hammady H, Fedorowicz Z, Elmagarmid A. Rayyan—a web and mobile app for systematic reviews. Syst Rev. 2016;5:210. doi: 10.1186/s13643-016-0384-4 27919275 PMC5139140

[39] Sterne JAC, Savović J, Page MJ, Elbers RG, Blencowe NS, Boutron I, et al. RoB 2: a revised tool for assessing risk of bias in randomised trials. BMJ. 2019;366:l4898. doi: 10.1136/bmj.l4898 31462531

[40] Wagner BD, Grunwald GK, Zerbe GO, Mikulich-Gilbertson SK, Robertson CE, Zemanick ET, et al. On the Use of Diversity Measures in Longitudinal Sequencing Studies of Microbial Communities. Front Microbiol. 2018;9:1037. doi: 10.3389/fmicb.2018.01037 29872428 PMC5972327

[41] Kim B-R, Shin J, Guevarra RB, Lee JH, Kim DW, Seol K-H, et al. Deciphering Diversity Indices for a Better Understanding of Microbial Communities. J Microbiol Biotechnol. 2017;27:2089–2093. doi: 10.4014/jmb.1709.09027 29032640

[42] Clarke KR, Somerfield PJ, Chapman MG. On resemblance measures for ecological studies, including taxonomic dissimilarities and a zero-adjusted Bray–Curtis coefficient for denuded assemblages. J Exp Mar Biol Ecol. 2006;330:55–80. doi: 10.1016/j.jembe.2005.12.017

[43] Doan T, Arzika AM, Ray KJ, Cotter SY, Kim J, Maliki R, et al. Gut Microbial Diversity in Antibiotic-Naive Children After Systemic Antibiotic Exposure: A Randomized Controlled Trial. Clin Infect Dis Off Publ Infect Dis Soc Am. 2017;64:1147–1153. doi: 10.1093/cid/cix141 28402408 PMC5849050

[44] Doan T, Hinterwirth A, Arzika AM, Cotter SY, Ray KJ, O’Brien KS, et al. Mass Azithromycin Distribution and Community Microbiome: A Cluster-Randomized Trial. Open Forum. Infect Dis. 2018;5:ofy182. doi: 10.1093/ofid/ofy182 30151409 PMC6101535

[45] Doan T, Hinterwirth A, Worden L, Arzika AM, Maliki R, Abdou A, et al. Gut microbiome alteration in MORDOR I: a community-randomized trial of mass azithromycin distribution. Nat Med. 2019;25:1370–1376. doi: 10.1038/s41591-019-0533-0 31406349

[46] Doan T, Worden L, Hinterwirth A, Arzika AM, Maliki R, Abdou A, et al. Macrolide and Nonmacrolide Resistance with Mass Azithromycin Distribution. N Engl J Med. 2020;383:1941–1950. doi: 10.1056/NEJMoa2002606 33176084 PMC7492079

[47] Hinterwirth A, Sié A, Coulibaly B, Ouermi L, Dah C, Tapsoba C, et al. Rapid Reduction of Campylobacter Species in the Gut Microbiome of Preschool Children after Oral Azithromycin: A Randomized Controlled Trial. Am J Trop Med Hyg. 2020;103:1266–1269. doi: 10.4269/ajtmh.19-0940 32524948 PMC7470541

[48] Oldenburg CE, Sié A, Coulibaly B, Ouermi L, Dah C, Tapsoba C, et al. Effect of Commonly Used Pediatric Antibiotics on Gut Microbial Diversity in Preschool Children in Burkina Faso: A Randomized Clinical Trial. Open Forum. Infect Dis. 2018;5:ofy289. doi: 10.1093/ofid/ofy289 30515431 PMC6262116

[49] Pickering H, Hart JD, Burr S, Stabler R, Maleta K, Kalua K, et al. Impact of azithromycin mass drug administration on the antibiotic-resistant gut microbiome in children: a randomized, controlled trial. Gut Pathog. 2022;14:5. doi: 10.1186/s13099-021-00478-6 34991704 PMC8740015

[50] Oldenburg CE, Hinterwirth A, Sié A, Coulibaly B, Ouermi L, Dah C, et al. Gut Resistome After Oral Antibiotics in Preschool Children in Burkina Faso: A Randomized, Controlled Trial. Clin Infect Dis Off Publ Infect Dis Soc Am. 2020;70:525–527. doi: 10.1093/cid/ciz455 31149703 PMC7456340

[51] Parker EPK, Praharaj I, John J, Kaliappan SP, Kampmann B, Kang G, et al. Changes in the intestinal microbiota following the administration of azithromycin in a randomised placebo-controlled trial among infants in south India. Sci Rep. 2017;7:9168. doi: 10.1038/s41598-017-06862-0 28835659 PMC5569098

[52] Zafar H, Saier MH. Gut Bacteroides species in health and disease. Gut Microbes. 13:1848158. doi: 10.1080/19490976.2020.1848158 33535896 PMC7872030

[53] Anthony WE, Wang B, Sukhum KV, D’Souza AW, Hink T, Cass C, et al. Acute and persistent effects of commonly used antibiotics on the gut microbiome and resistome in healthy adults. Cell Rep. 2022;39:110649. doi: 10.1016/j.celrep.2022.110649 35417701 PMC9066705

[54] Harris VC, Haak BW, Handley SA, Jiang B, Velasquez DE, Hykes BL, et al. Effect of Antibiotic-Mediated Microbiome Modulation on Rotavirus Vaccine Immunogenicity: A Human Randomized-Control Proof-of-Concept Trial. Cell Host Microbe. 2018;24:197–207.e4. doi: 10.1016/j.chom.2018.07.005 30092197 PMC11514417

[55] Roubaud-Baudron C, Ruiz VE, Swan AM, Vallance BA, Ozkul C, Pei Z, et al. Long-Term Effects of Early-Life Antibiotic Exposure on Resistance to Subsequent Bacterial Infection. mBio. 2019;10:e02820–e02819. doi: 10.1128/mBio.02820-19 31874917 PMC6935859

[56] van Nood E, Vrieze A, Nieuwdorp M, Fuentes S, Zoetendal EG, de Vos WM, et al. Duodenal Infusion of Donor Feces for Recurrent Clostridium difficile. N Engl J Med. 2013;368:407–415. doi: 10.1056/NEJMoa1205037 23323867

[57] Brandl K, Plitas G, Mihu CN, Ubeda C, Jia T, Fleisher M, et al. Vancomycin-resistant enterococci exploit antibiotic-induced innate immune deficits. Nature. 2008;455:804–807. doi: 10.1038/nature07250 18724361 PMC2663337

[58] Do NTT, Vu HTL, Nguyen CTK, Punpuing S, Khan WA, Gyapong M, et al. Community-based antibiotic access and use in six low-income and middle-income countries: a mixed-method approach. Lancet Glob Health. 2021;9:e610–e619. doi: 10.1016/S2214-109X(21)00024-3 33713630 PMC8050200

[59] Lynch SV, Pedersen O. The Human Intestinal Microbiome in Health and Disease. N Engl J Med. 2016;375:2369–2379. doi: 10.1056/NEJMra1600266 27974040

[60] Cox LM, Yamanishi S, Sohn J, Alekseyenko AV, Leung JM, Cho I, et al. Altering the intestinal microbiota during a critical developmental window has lasting metabolic consequences. Cell. 2014;158:705–721. doi: 10.1016/j.cell.2014.05.052 25126780 PMC4134513

[61] Borbet TC, Pawline MB, Zhang X, Wipperman MF, Reuter S, Maher T, et al. Influence of the early-life gut microbiota on the immune responses to an inhaled allergen. Mucosal Immunol. 2022;15:1000–1011. doi: 10.1038/s41385-022-00544-5 35842561 PMC9835105

[62] Bell T, Newman JA, Silverman BW, Turner SL, Lilley AK. The contribution of species richness and composition to bacterial services. Nature. 2005;436:1157–1160. doi: 10.1038/nature03891 16121181

[63] University of California, San Francisco. Evaluating Impact of Azithromycin Mass Drug Administrations on All-cause Mortality and Antibiotic Resistance: Mortality Trial. clinicaltrials.gov; 2020 Aug. Report No.: NCT02047981. Available from: https://clinicaltrials.gov/ct2/show/NCT02047981.

[64] University of California, San Francisco. Antibiotic Resistance and Microbiome in Children Aged 6–59 Months in Nouna, Burkina Faso. clinicaltrials.gov; 2021 May. Report No.: NCT03187834. Available from: https://clinicaltrials.gov/ct2/show/NCT03187834.

[65] Kwon Y, Cho Y-S, Lee Y-M, Kim S, Bae J, Jeong S-J. Changes to Gut Microbiota Following Systemic Antibiotic Administration in Infants. Antibiotics. 2022;11:470. doi: 10.3390/antibiotics11040470 35453221 PMC9025670

[66] Wei S, Mortensen MS, Stokholm J, Brejnrod AD, Thorsen J, Rasmussen MA, et al. Short- and long-term impacts of azithromycin treatment on the gut microbiota in children: A double-blind, randomized, placebo-controlled trial. EBioMedicine. 2018;38:265–272. doi: 10.1016/j.ebiom.2018.11.035 30478001 PMC6306380

[67] Bai L, Zhou P, Li D, Ju X. Changes in the gastrointestinal microbiota of children with acute lymphoblastic leukaemia and its association with antibiotics in the short term. J Med Microbiol. 2017;66:1297–1307. doi: 10.1099/jmm.0.000568 28855025

[68] Reyman M, van Houten MA, Watson RL, Chu MLJN, Arp K, de Waal WJ, et al. Effects of early-life antibiotics on the developing infant gut microbiome and resistome: a randomized trial. Nat Commun. 2022;13:893. doi: 10.1038/s41467-022-28525-z 35173154 PMC8850541

