# Supplementary figures and images for: Impact of antibiotics on gut microbiome composition and resistome in the first years of life in low- to middle-income countries: A systematic review
Source: PLoS Med. 2023 Jun 27;20(6):e1004235. doi: 10.1371/journal.pmed.1004235 (PMC10298773; doi:10.1371/journal.pmed.1004235)

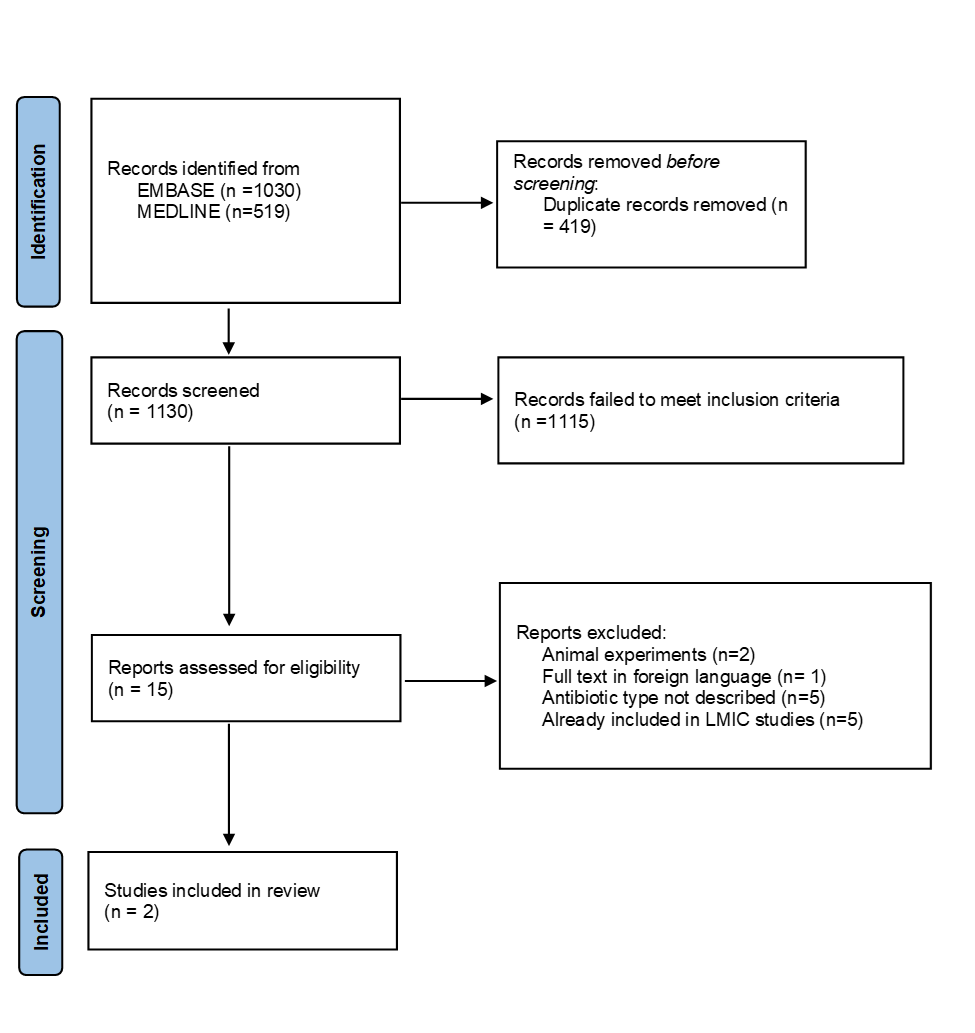

Supplement: S1 Fig — Results of searches from 2021 to August 2022 in high-income countries. (TIF) [file pmed.1004235.s002.tif]

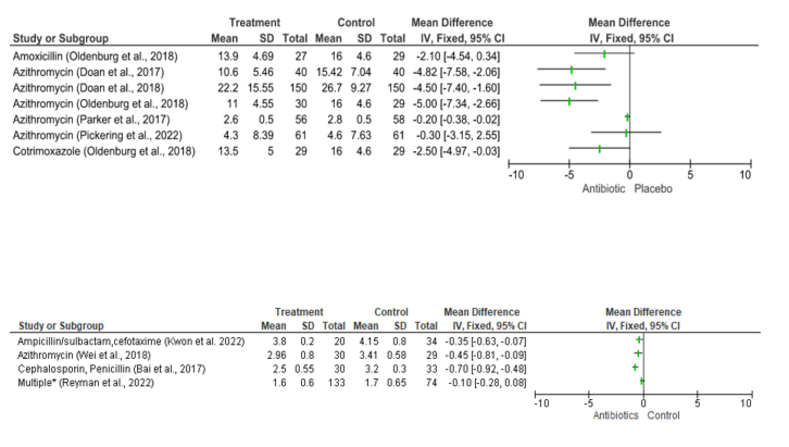

Supplement: S2 Fig — Multiple* refers to penicillin + gentamicin, amoxicillin+clavulanic acid + gentamicin, or amoxicillin + cefotaxime. Doan and colleagues (2017) [43], Doan and colleagues (2018) [44], Oldenburg and colleagues (2018) [48], Pickering and colleagues (2022) [49], Parker and colleagues (2017) [51], Kwon and colleagues (2020) [65], Wei and colleagues (2018) [66], Bai and colleagues (2017) [67], Reyman and colleagues (2022) [68]. (TIF) [file pmed.1004235.s003.tif]

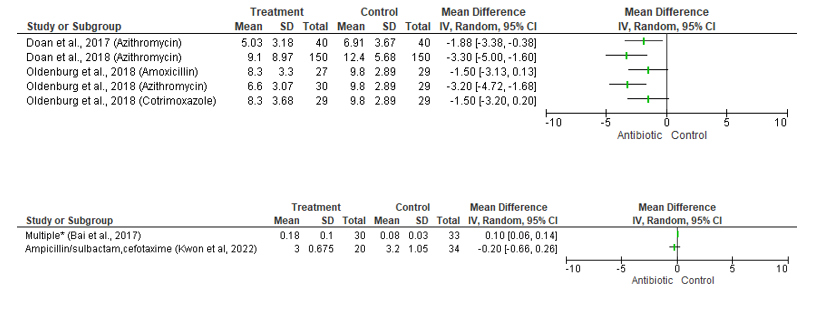

Supplement: S3 Fig — Multiple* refers to penicillin + gentamicin, co-amoxiclav + gentamicin or amoxicillin + cefotaxime. Doan and colleagues (2017) [43], Doan and colleagues (2018) [44], Oldenburg and colleagues (2018) [48], Kwon and colleagues (2020) [65], Bai and colleagues (2017) [67]. (TIF) [file pmed.1004235.s004.tif]

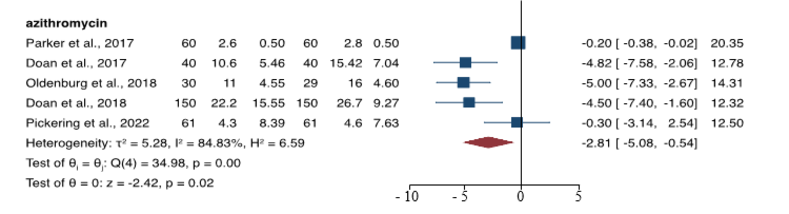

Supplement: S4 Fig — Estimates were obtained using random effects restricted maximum likelihood model. Doan and colleagues (2017) [43], Doan and colleagues (2018) [44], Oldenburg and colleagues (2018) [48], Pickering and colleagues (2022) [49], Parker and colleagues (2017) [51]. (TIF) [file pmed.1004235.s005.tif]
