# Supplementary material for: Impact of antibiotics on gut microbiome composition and resistome in the first years of life in low- to middle-income countries: A systematic review
Source: PLoS Med. 2023 Jun 27;20(6):e1004235. doi: 10.1371/journal.pmed.1004235 (PMC10298773; doi:10.1371/journal.pmed.1004235)
Supplement: S1 Text — (DOCX) [file pmed.1004235.s009.docx]

**Impact of antibiotics on gut microbiome composition and resistome in the first years of life in low- to middle-income countries: A systematic review**

**Authors**: Charlie C Luchen^1,2^, Mwelwa Chibuye^1,2^, Rene Spijker^1^, Michelo Simuyandi^2^, Caroline Chisenga^2^, Samuel Bosomprah^2,3^, Roma Chilengi^2,4,5^, Constance Schultsz^1,6,7^, Daniel R Mende^6,7^, Vanessa Harris^1,7,8*^

**Supporting Information**

Supporting Text: Systematic review for High Income Countries

Supporting Table 4**:** Overview of included studies from HIC

**Supporting Text**

**Study results for high-income countries**

Study Characteristics

4 studies were identified and included in the secondary HIC analysis (S4 Table). The studies were performed in the Netherlands, China, Korea, and Denmark. The studies had varied study designs namely: cohort (1), cross sectional (1), and RCT (2). Whereas studies performed in LMIC included only healthy participants [1–5] those from HIC were hospitalized for fever [6], asthma-like symptoms [7], suspected early onset neonatal sepsis [8] and healthy participants [9]. The majority of HIC studies investigated a combined effect of more than one antibiotic on the gut microbiome diversity including the effects of a 3 day course of oral cephalosporin and a 10 day course of intravenous penicillin or cephalosporins [9], 48 hours or treatment with intravenous penicillin + gentamicin, amoxicillin with clavulanic acid + gentamicin, or amoxicillin + cefotaxime [8], and the effects of a 3 day course of intravenous ampicillin/sulbactam [6]. One HIC study assessed these mean difference changes in alpha diversity following one course of azithromycin [7].

Supporting Table 4. Overview of included studies from HIC

| Author | Setting | Design | sample size | exposure | Duration of course in days | | Time from treatment to analysis in days | Sequencing | findings |  |
| --- | --- | --- | --- | --- | --- | --- | --- | --- | --- | --- |
| Kwon, 2022 | Korea | cohort | 54 | ampicillin/sulbactam, cefotaxime (all iv) | | 3 | 28 | 16S rRNA | α diversity decreased, significant reduction in Shannon diversity in treatment arm mean compared to placebo | |
| Bai, 2017 | China | Cross sectional | 63 | cephalosporin iv/po, penicillin iv | | 10 | n/a | 16S rRNA | α diversity decreased, significant reduction in Shannon diversity and Simpson diversity in treatment arm compared to placebo mean | |
| Wei, 2018 | Denmark | RCT | 72 | azithromycin po | | 3 | 11 | 16S rRNA | α diversity decreased, nonsignificant reduction in Shannon diversity in azithromycin group compared to the placebo | |
| Reyman, 2022 | Netherlands | RCT | 147 | penicillin + gentamicin, co-amoxiclav + gentamicin or amoxi + cefotaxime (all iv) | | 1 | 365 | 16S rRNA | α diversity decreased, nonsignificant reduction in Shannon diversity in treatment arm compared to placebo | |

Abbreviations: iv, intravenous; po, *per os* (oral); amoxi, amoxicillin; amoxiclav, amoxicillin with clavulanic acid

*Differential impact of antibiotics in LMIC vs HIC*

They were notable differences in the LMICs vs HICs study design and methodology limiting comparability. Possible cofounders in the comparison include differences in health status and duration of antibiotic exposure between LMIC and HIC. LMICs participants were predominately healthy children and HICs participants had underlying conditions.

To understand if antibiotics have similar impacts on microbiome diversity and composition between infants from HIC and LMIC, we carried out a comparison of the mean differences in alpha diversity between studies carried out in LMICs and HICs. We report the effect sizes for each study separately as the studies had noticeable methodological differences (e.g. reports at different taxonomic levels). Given the combined treatment design of some of the studies performed in HIC, it was not possible to report individual antibiotic effects on alpha diversity as was done with LMIC, which were all randomized controlled trials.

The majority of LMIC studies showed a significant reduction of mean alpha diversity in the treatment arm compared to the control regardless of antibiotic used when measured by the Shannon index (5 out of 7 studies, S2 Fig) or Simpson’s index (3 out of 5 studies, S3 Fig). In parallel, two of the HIC studied showed statistically significant reductions in mean alpha diversity between antibiotic treatment and control with visible trends towards lower Shannon diversity in all studies. The magnitude of mean difference in Shannon diversity was smaller for HIC than LMIC studies.

**Discussion**

This systematic review was unable to identify sufficient studies to permit proper comparison of the impact of antimicrobials across HIC and LMIC regions. LMIC studies in this review were conducted in predominantly healthy children while studies conducted with HIC infants had concomitant illnesses. Nevertheless, we were able to show consistent trends on the effects of antibiotic use on the microbiome diversity in both regions. Antibiotics, regardless of class, generally resulted in a diminished microbiome diversity in the treatment arms as compared to the controls. In the limited number of studies identified in this review, the effect size of this reduction in diversity tended to be larger in LMICs as compared to HIC. Two possible explanations could account for this difference. One explanation could be that infants in HIC studies already had a less diverse microbiome before treatment due to illness, resulting in a less pronounced antibiotic treatment effect when compared to control groups. Another possible explanation is that baseline alpha diversity may be higher in LMIC settings than HIC settings in infants, as previously reported for Western and African populations [11]. Hence these baseline differences in diversity could play a role in the observed mean differences in effect size regardless of health status.

**References**

1. Doan T, Arzika AM, Ray KJ, Cotter SY, Kim J, Maliki R, et al. Gut Microbial Diversity in Antibiotic-Naive Children After Systemic Antibiotic Exposure: A Randomized Controlled Trial. Clin Infect Dis Off Publ Infect Dis Soc Am. 2017;64: 1147–1153. doi:10.1093/cid/cix141

2. Doan T, Hinterwirth A, Arzika AM, Cotter SY, Ray KJ, O’Brien KS, et al. Mass Azithromycin Distribution and Community Microbiome: A Cluster-Randomized Trial. Open Forum Infect Dis. 2018;5: ofy182. doi:10.1093/ofid/ofy182

3. Oldenburg CE, Sié A, Coulibaly B, Ouermi L, Dah C, Tapsoba C, et al. Effect of Commonly Used Pediatric Antibiotics on Gut Microbial Diversity in Preschool Children in Burkina Faso: A Randomized Clinical Trial. Open Forum Infect Dis. 2018;5: ofy289. doi:10.1093/ofid/ofy289

4. Parker EPK, Praharaj I, John J, Kaliappan SP, Kampmann B, Kang G, et al. Changes in the intestinal microbiota following the administration of azithromycin in a randomised placebo-controlled trial among infants in south India. Sci Rep. 2017;7: 9168. doi:10.1038/s41598-017-06862-0

5. Pickering H, Hart JD, Burr S, Stabler R, Maleta K, Kalua K, et al. Impact of azithromycin mass drug administration on the antibiotic-resistant gut microbiome in children: a randomized, controlled trial. Gut Pathog. 2022;14: 5. doi:10.1186/s13099-021-00478-6

6. Kwon Y, Cho Y-S, Lee Y-M, Kim S, Bae J, Jeong S-J. Changes to Gut Microbiota Following Systemic Antibiotic Administration in Infants. Antibiotics. 2022;11: 470. doi:10.3390/antibiotics11040470

7. Wei S, Mortensen MS, Stokholm J, Brejnrod AD, Thorsen J, Rasmussen MA, et al. Short- and long-term impacts of azithromycin treatment on the gut microbiota in children: A double-blind, randomized, placebo-controlled trial. EBioMedicine. 2018;38: 265–272. doi:10.1016/j.ebiom.2018.11.035

8. Reyman M, van Houten MA, Watson RL, Chu MLJN, Arp K, de Waal WJ, et al. Effects of early-life antibiotics on the developing infant gut microbiome and resistome: a randomized trial. Nat Commun. 2022;13: 893. doi:10.1038/s41467-022-28525-z

9. Bai L, Zhou P, Li D, Ju X 2017. Changes in the gastrointestinal microbiota of children with acute lymphoblastic leukaemia and its association with antibiotics in the short term. J Med Microbiol. 66: 1297–1307. doi:10.1099/jmm.0.000568

10. Chokshi A, Sifri Z, Cennimo D, Horng H. Global Contributors to Antibiotic Resistance. J Glob Infect Dis. 2019;11: 36–42. doi:10.4103/jgid.jgid_110_18

11. Yatsunenko T, Rey FE, Manary MJ, Trehan I, Dominguez-Bello MG, Contreras M, et al. Human gut microbiome viewed across age and geography. Nature. 2012;486: 222–227. doi:10.1038/nature11053
